# The cortical spectrum: A robust structural continuum in primate cerebral cortex revealed by histological staining and magnetic resonance imaging

**DOI:** 10.3389/fnana.2022.897237

**Published:** 2022-09-09

**Authors:** Yohan J. John, Basilis Zikopoulos, Miguel Ángel García-Cabezas, Helen Barbas

**Affiliations:** ^1^Neural Systems Laboratory, Department of Health Sciences, Boston University, Boston, MA, United States; ^2^Human Systems Neuroscience Laboratory, Department of Health Sciences, Boston University, Boston, MA, United States; ^3^Graduate Program in Neuroscience, Boston University School of Medicine, Boston, MA, United States; ^4^Department of Anatomy and Neurobiology, Boston University School of Medicine, Boston, MA, United States

**Keywords:** anatomy, cortex, neuroimaging, MRI, myelin, SMI-32, T1/T2, primate

## Abstract

High-level characterizations of the primate cerebral cortex sit between two extremes: on one end the cortical mantle is seen as a mosaic of structurally and functionally unique areas, and on the other it is seen as a uniform six-layered structure in which functional differences are defined solely by extrinsic connections. Neither of these extremes captures the crucial neuroanatomical finding: that the cortex exhibits systematic gradations in architectonic structure. These gradations have been shown to predict cortico-cortical connectivity, which in turn suggests powerful ways to ground connectomics in anatomical structure, and by extension cortical function. A challenge to widespread use of this concept is the labor-intensive and invasive nature of histological staining, which is the primary means of recognizing anatomical gradations. Here we show that a novel computational analysis technique can provide a coarse-grained picture of cortical variation. For each of 78 cortical areas spanning the entire cortical mantle of the rhesus macaque, we created a high dimensional set of anatomical features derived from captured images of cortical tissue stained for myelin and SMI-32. The method involved semi-automated de-noising of images, and enabled comparison of brain areas without hand-labeling of features such as layer boundaries. We applied multidimensional scaling (MDS) to the dataset to visualize similarity among cortical areas. This analysis shows a systematic variation between weakly laminated (limbic) cortices and sharply laminated (eulaminate) cortices. We call this smooth continuum the “cortical spectrum”. We also show that this spectrum is visible within subsystems of the cortex: the occipital, parietal, temporal, motor, prefrontal, and insular cortices. We compared the MDS-derived spectrum with a spectrum produced using T1- and T2-weighted magnetic resonance imaging (MRI) data derived from macaque, and found close agreement of the two coarse-graining methods. This suggests that T1w/T2w data, routinely obtained in human MRI studies, can serve as an effective proxy for data derived from high-resolution histological methods. More generally, this approach shows that the cortical spectrum is robust to the specific method used to compare cortical areas, and is therefore a powerful tool to understand the principles of organization of the primate cortex.

## Introduction

Among descriptions of cortical architecture, two diametrically opposed pictures can be posited. At one extreme, the cortex is presented as a patchwork quilt of distinct areas separated by defined boundaries. The century-old architectonic map of Korbinian Brodmann can create this impression: the order of area numbers is arbitrary, conveying no information about similarities and differences of architectonic structure (Brodmann, 1909). At the other extreme, the entire cortex is presented as a uniform structure (Rockel et al., 1980; Carlo and Stevens, 2013), which is often described in terms of canonical columns or microcolumns [reviewed in Horton and Adams (2005)]. This perspective can create the impression that cortical areas are equivalent, modular computational units, and that differences in function arise solely from differences in extrinsic connectivity. Both pictures of cortical structure are misleading. Further, using cytoarchitectonic features to divide the cortex into discrete areas has not led to consensus: researchers have not agreed on consistent criteria for drawing areal boundaries, which are often subtle (Campbell, 1905; Brodmann, 1909; von Economo et al., 1925; von Economo, 1927).

On the other hand, evidence that cortical architecture is not uniform across areas (Collins et al., 2010) has provided the basis to observe that the cellular and laminar features of areas vary systematically, so that each area is not totally discontinuous from its neighbors [reviewed in Sanides (1970), Barbas (2015), García-Cabezas et al. (2019, 2020)]. Following the approach of von Economo et al. (1925) and von Economo (1927), investigators began classifying cortical areas according to broad types, based on laminar features that vary in a graded manner, such as the number and prominence of layers and the sharpness of transitions between layers. Here we refer to this gradation of the cortical layering as the “degree of lamination.”

Several structural features appear to co-vary with the gradient of laminar elaboration (Pandya et al., 1988; Palomero-Gallagher and Zilles, 2018). Cortical types must be understood as discretizations of these continuous gradients. The number of cortical types depends on pragmatic considerations, and can vary from three to as many as eight (Hilgetag et al., 2016), but their ordering, based on degree of lamination, and their topological arrangement are not arbitrary, allowing for comparison among typologies.

The one-dimensional gradient of degree of lamination has proven to be an accurate indicator of cortico-cortical connectivity, enabling prediction of the pattern of laminar projections from one area to another. The linkage of connections to the systematic variation of the cortex, known as the Structural Model (Barbas, 1986; Barbas and Rempel-Clower, 1997), has been validated for all cortico-cortical areas thus far studied, and offers a unified framework for studying cortical structure, function, development, and evolutionary origin [reviewed in Barbas (2015) and García-Cabezas et al. (2019)].

Determining cortical type by examination of Nissl-stained sections (García-Cabezas et al., 2020) requires training in neuroanatomy, and retains an element of subjectivity. To avoid these challenges, quantitative proxies for degree of lamination have been used, such as overall neural density (Dombrowski et al., 2001; Cahalane et al., 2012; Hilgetag et al., 2016). However, estimating neural density is a labor-intensive process that has not been automated yet, and it is not always the best proxy for cortical type. For example, motor cortex is a eulaminate cortex but has relatively low neural density compared to other eulaminate areas (García-Cabezas and Barbas, 2014). It is therefore necessary to develop coarse-grained estimates of cortical structure that can be used by researchers without extensive training in neuroanatomy. A coarse-grained synopsis of cortical structure can provide a bird’s eye view that is essential for identifying systematic structural patterns. Further, as new histological and gene expression approaches are introduced (Herculano-Houzel et al., 2013; Palomero-Gallagher and Zilles, 2018), they can be incorporated into this synoptic method.

Here we used a novel computational procedure applied to analysis of photomicrographs of cortical areas using cellular and axonal architectonic markers. From these photomicrographs we computed a dataset of cortical profiles of 78 areas in the rhesus macaque brain. To reduce the dimensionality of this dataset for the purposes of visualization and interpretation, we performed multidimensional scaling (MDS). This enabled an integrated view of structural similarities and differences among cortical areas. This approach allowed visualization of the systematic gradation in structure across the entire cortex.

We also showed that the same gradation, which we call the “cortical spectrum,” is reflected in magnetic resonance imaging (MRI) data, which are collected broadly in humans. Specifically, we contrasted our architectonic results with analysis of rhesus macaque MRI data. For humans, MRI-based methods are now the most widespread means of determining anatomical structure (Glasser et al., 2016). These methods are also being used in non-human primates (Bock et al., 2009; Large et al., 2016; Vickery et al., 2020). We show that T1- and T2-weighted MRI signals provide qualitative results that are in agreement with the more high-resolution analysis based on stained tissue. This raised the possibility that MRI analysis, which can be challenging to interpret, may serve as a proxy for architectonic analysis, and more specifically for inferring the degree of lamination in the cortex in humans using non-invasive methods.

## Materials and methods

**FIGURE 1:**
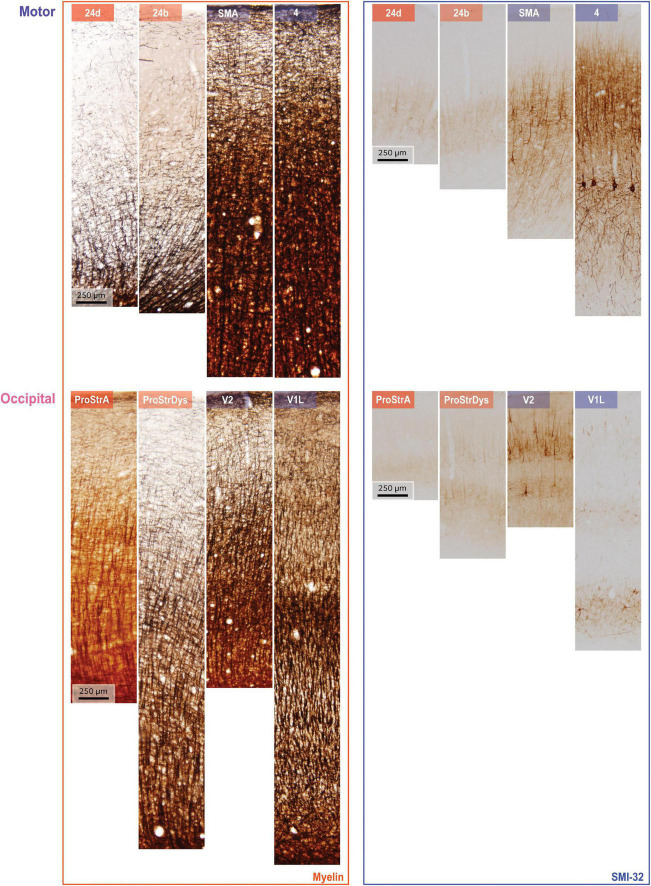
Examples of myelin staining (left column) and SMI-32 staining (right column). Rows show data from two systems, arranged in order from limbic (left) to eulaminate (right). Top: areas along the motor cortical axis, bottom: areas along the occipital cortical axis. Variations in cortical thickness from pia to white matter, for both myelin and SMI-32, arise due to non-uniform shrinkage during tissue processing.

We used coronal brain sections from two rhesus macaque monkeys (cases AN and AQ) to analyze a total of 78 cortical regions, from one hemisphere in each case. These areas span the entire cortex, but are not an exhaustive sample of structural gradation, so the smooth gradation in structure is not fully reflected for every region. These cases have been used in other unrelated studies (e.g., García-Cabezas et al., 2017; Zikopoulos et al., 2018), so here we will describe a brief overview of tissue processing. Perfusion and sectioning of the brains were performed as reported previously (Barbas and Rempel-Clower, 1997; Zikopoulos et al., 2018). Experiments were performed in accordance with the National Institutes of Health Guide for the care and use of laboratory animals (publication 80–22 revised, 1996). Protocols were approved by the Institutional Animal Care and Use Committee at Boston University School of Medicine, Harvard Medical School, and New England Primate Research Center. Tissue sections were 40 micrometers (μm) thick. For each case, one set of sections was stained for myelin (Gallyas), and a matched set was stained for SMI-32, an antibody to a neurofilament protein that labels a subset of pyramidal projection neurons in layers II-III and V-VI (Campbell and Morrison, 1989; Hof et al., 1995). Sections were photographed at 4 × 10× magnification. These photomicrographs were then rotated and cropped to highlight a segment extending from the pia to the superficial white matter. Cropping was performed with the help of matched Nissl-stained sections, so as to include layer six and a minimal part of superficial white matter. Figure 1 shows examples of such photomicrographs for two cortical systems in the brain that include areas in motor and occipital cortices. The vertical height of each photograph varied depending on the cortical thickness. Widths also varied, as cropping was constrained by the amount of cortical curvature. Each cropped color image was first converted to grayscale using Matlab’s “rgb2gray” function, and then inverted: each pixel’s gray value was subtracted from 255 to create a gray level. Thus, the pixel values varied in proportion to the darkness of the stain.

A key challenge with histological staining methods is variability of baseline density of staining from case to case, and also of overall staining across sections within a case. In addition, staining artifacts can arise in experiments. Classically, when such sections are analyzed, the noise and variability are filtered out through subjective discernment of the relevant qualitative features. This skilled filtering has proved difficult to automate with machine learning, given the absence of vast hand-labeled datasets. For myelin-stained sections, the key qualitative features include the density of labeled myelin fibers, the presence and spacing of fiber bands, and the extent of laminar penetration of fibers. For SMI-32-stained sections, the qualitative features include the presence, size and laminar specificity of labeled neurons.

**FIGURE 2:**
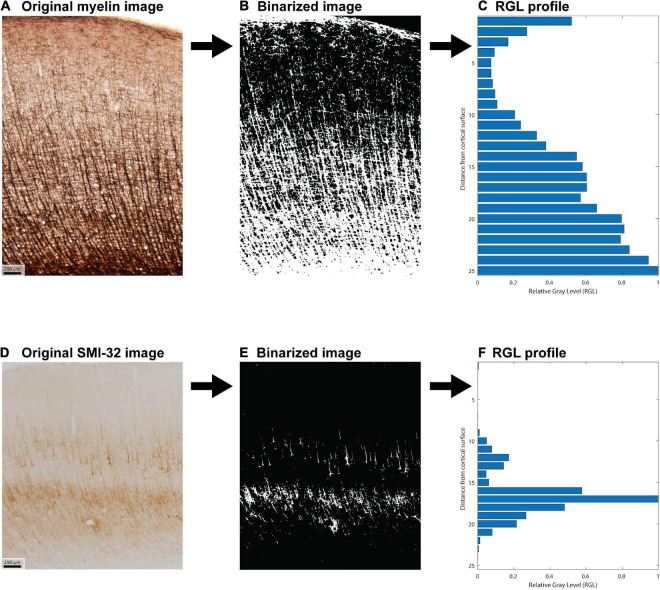
Example of image processing used to create the feature vector for each cortical area. **(A)** Original photomicrograph of myelin stain of area 46 dorsal. **(B)** Corresponding binarized image. **(C)** Relative gray level (RGL) profile generated from the binarized image. **(D–F)** Analogous steps for a photomicrograph of SMI-32 stain of area 46 dorsal.

Hand-labeled datasets, which are necessary for subsequent quantitative stereological estimation, are impractical if the goal is to study all cortical areas together. We therefore developed a semi-automated method to extract coarse-grained features from a large number of cortical areas (Figure 2). We first processed each image to generate binarized “all-or-nothing” labeled pixels (Figures 2B,E). We determined a threshold for each stained image: pixels above the threshold were assigned a non-zero value of unity. To estimate these thresholds in an unbiased fashion, we presented the images to three researchers in pseudorandom order, with the area labels withheld. We instructed the researchers to adjust the thresholds in order to balance a trade-off between the architectonic features and the noise, background, and staining artifacts. We designed custom software for this purpose, in which the original image was shown side-by-side with the thresholded binarized image. The researchers were instructed to step through the images and use a slider to adjust the threshold for each. For a given cortical area in a case, the threshold for a given stain type, myelin or SMI-32, was averaged from the estimates made by the researchers. The binarized images were used to compute cortical profiles. The researchers’ thresholds showed reasonable agreement, as assessed by a measure of inter-rater reliability (Krippendorff’s alpha = 0.71).

For each binarized image, we computed a cortical profile by dividing the image into equal-sized sub-images along the direction normal to the surface (the pia-to-white-matter direction) and averaging the pixel intensity within each sub-image or bin. These profiles were then normalized by the largest bin value. This resulted in a relative gray level (RGL) profile, which captures the relative variation of the stain with depth from the cortical surface (Figures 2C,F). Analyses were conducted for binarized images cropped to be of equal width, as well as for the uncropped binarized images. Results were qualitatively similar in both cases, so the results shown here are from the analysis of the uncropped images. We divided each image into 25 bins, which exceeds the standard number of layers commonly used to analyze cortical structure. Figure 2 shows an example of the image processing stages for area 46 dorsal (case AN).

The 25-dimensional RGL profiles from the myelin and SMI-32 images were concatenated to create a 50-dimensional feature vector for each cortical area. Each of the 78 feature vectors was the result of averaging feature vectors across the two cases. This averaged feature set was used to create a difference matrix using the L2 norm, which served as the basis for multidimensional scaling (MDS). This method enables high-dimensional data points to be mapped into a lower dimensional space that preserves the differences among the points. A small number of dimensions, three in this case, facilitates visualization of the differences among cortical areas. The feature set was z-scored and then used to create a difference matrix among the 78 brain areas. For the comparison of different coarse-grained methods, we also computed the average RGL values for each binarized image. For comparison, cortical areas were classified by experts (BZ and MG-C) using matched Nissl-stained sections, in accordance with previously published criteria (Pandya et al., 1988; García-Cabezas et al., 2020).

Image rotation and cropping was performed using ImageJ. Further processing was performed using MATLAB (R2021b). MDS was performed using MATLAB’s in-built function. To study the robustness of the qualitative results, we also performed a principal component analysis (PCA) on the same dataset (Supplementary material). The accuracy of the MDS dimensions for capturing inter-area differences was assessed using linear regression: the Euclidean differences (L2 norm) between each pair of cortical areas in MDS-space were regressed on the corresponding differences given by the original Z-scored dataset. Statistical analyses were performed using Matlab’s in-built correlation (“corr”) and partial correlation (“partialcorri”) functions. A Matlab package was used to calculate Krippendoff’s alpha (Jana, 2022).

For the analysis of MRI data, we used the T1-weighted (T1w) and T2-weighted (T2w) datasets of a shared adult rhesus macaque MRI scan from McGill University obtained from the NeuroImaging Tools & Resources Collaboratory (www.nitrc.org), and we estimated the T1w/T2w ratio. The scan was taken from a living subject. The dataset is available for download^1^ under the Creative Commons—Attribution-Non-Commercial Share Alike (CC-BY-NC-SA)-Standard INDI data sharing policy, which prohibits use of the data for commercial purposes. A Siemens 3 Tesla Trio scanner with a custom 8-channel phased-array receive coil with the standard Siemens Trio shimming optimization of the magnetic field prior to data acquisition was used for the structural scans. The T1 MP2RAGE and the T2 SPACE sequences had a voxel resolution of 0.6 mm, isotropic, with the following additional parameters [for T1: 3.65 ms (TE), 5,000 ms (TR), 700 and 2,500 ms (TI), 4° and 5° (Flip angles), 242 mm × 227 mm × 106 mm (FOV read), and 176 slices; for T2: 320 ms (TE), 3,200 ms (TR), 120° (Flip angle), two averages, 116 mm × 116 mm × 77 mm (FOV read), and 128 slices]. The T1w/T2w ratio reflects the content of intracortical myelin, and is widely used in imaging studies of cortical hierarchies and connectivity (Glasser et al., 2016; Zhang et al., 2020). T1 and T2 sequences were resliced at the coronal level using ImageJ and individual slices were manually inspected and matched with the most similar histological sections that included the ROIs analyzed using histology or immunohistochemistry. For the estimation of mean gray value (optical density) matching ROIs from the cortical surface to the white matter were selected in coronal plane images from each of the 78 cortical areas. Due to the low MRI resolution, compared to the high resolution of the histological sections, reliable layer distinction was not feasible (Supplementary Figure 3). We therefore estimated one mean gray optical density value for the entire depth of the gray matter (cortical thickness ranging from 1 to 4 voxels) per area in each hemisphere.

The T1w/T2w ratio reflects the content of intracortical myelin, and is widely used in imaging studies of cortical hierarchies and connectivity (Glasser et al., 2016; Zhang et al., 2020).

## Results

We used sections stained for myelin- and SMI-32- to capture and analyze photomicrographs from two rhesus macaque brains to create a combined dataset consisting of the myelin and SMI-32 profiles of 78 cortical areas. We used this high-dimensional dataset as the basis for MDS analysis, which allowed us to transform high-dimensional cortical data into a three-dimensional space that preserved the relative differences among cortical areas. MDS reflects the differences among areas in the original dataset accurately, suggesting a good agreement between the high-dimensional dataset and the derived three-dimensional space. We assessed the accuracy of the MDS dimensions for capturing inter-areal differences using linear regression. The slope of the regression line was 1.05 and the adjusted R-squared value was 0.84, indicative of a good fit (Supplementary Figure 2).

**FIGURE 3:**
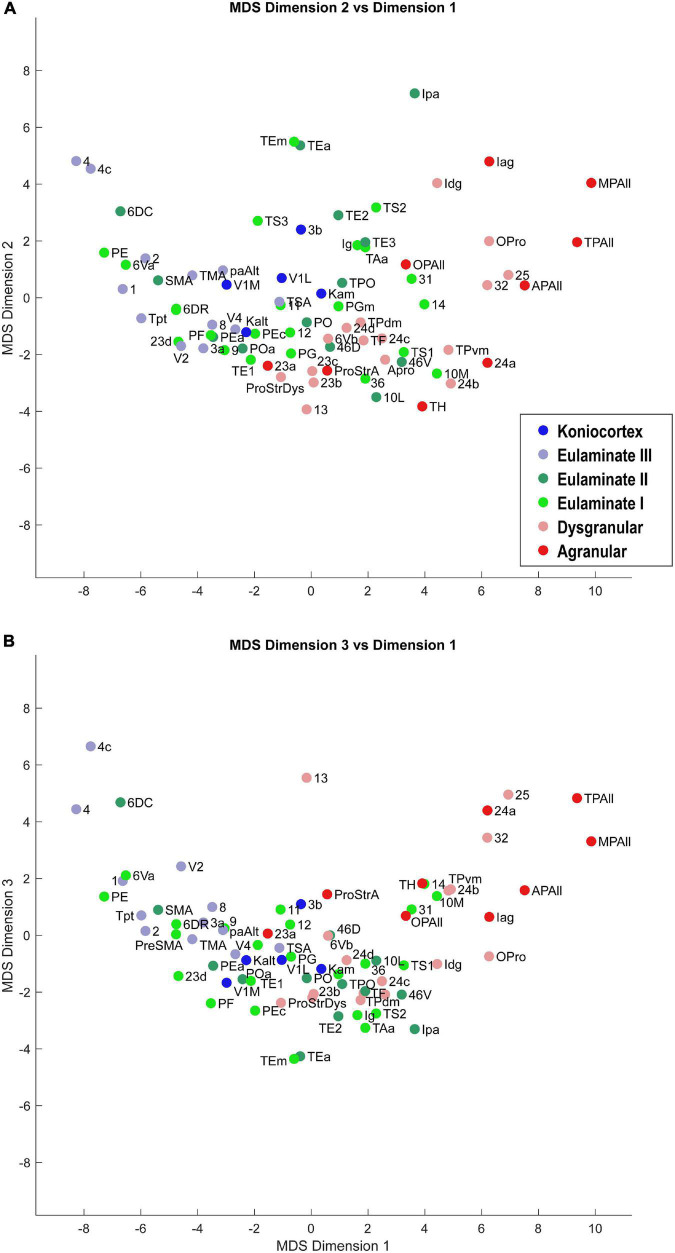
Scatter plot of 78 cortical areas in MDS-derived dimensions. We used three dimensions for MDS. Dimension 1 showed the clearest alignment with the subjective classification of cortical types according to degree of lamination. **(A)** Dimension 2 versus Dimension 1. **(B)** Dimension 3 versus Dimension 1. Due to partial overlap of data points, some area labels were omitted for clarity.

**FIGURE 4:**
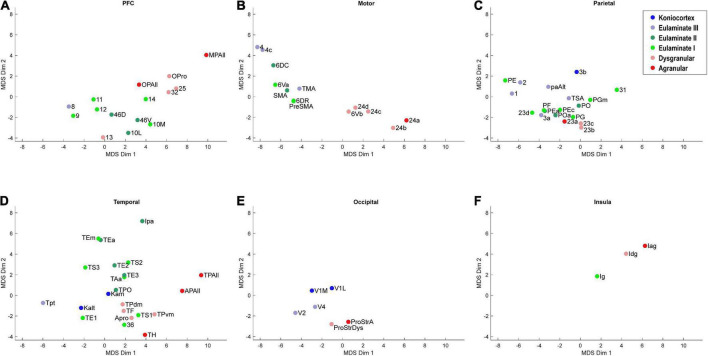
Scatter plot of 78 cortical areas in MDS-derived dimensions, divided by cortical system. Only the first two dimensions are plotted. **(A)** Prefrontal cortex, **(B)** Motor cortex, **(C)** Parietal cortex, **(D)** Temporal cortex, **(E)** Occipital cortex, **(F)** Insular cortex. Due to overlap of data points, area labels were omitted from some of the points.

Dimensionality reduction allowed us to visualize the relationships of similarity and difference among the cortical areas. Scatter plots of the cortical areas in the MDS-space showed a clear gradation of cortical areas: the less sharply laminated limbic areas were at one end of the continuum, and the more sharply laminated primary sensory and motor areas were at the other end (Figures 3, 4). The data suggest that cortical areas do not separate into distinct clusters, but show graded variation. These results show that the cortex can be described as a structural spectrum.

**FIGURE 5:**
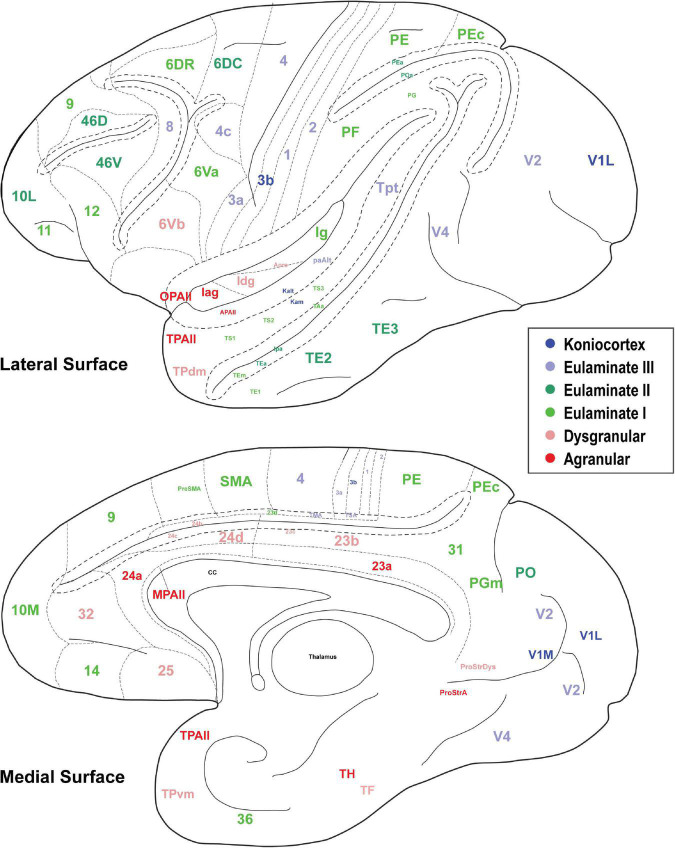
The cortical spectrum. Seventy eight brain areas used in the analyses. Colors correspond to the subjective categorization performed by experts. Title location reflects approximate center of region.

In Figures 3, 4, the cortical areas were shown using color-coded text that represents the subjective categorization by experts based on previously published criteria (Pandya et al., 1988; García-Cabezas et al., 2020). This categorization is overlaid on a map of the cortex in Figure 5. We divided the 78 cortical areas into six discrete levels of lamination: agranular (red), dysgranular (pink), eulaminate I (light green), eulaminate II (dark green), eulaminate III (light blue), and koniocortex (dark blue). Agranular (lacking a granular layer IV) and dysgranular (having a weak or incipient layer IV) are collectively referred to as limbic cortices. The non-limbic cortices are collectively described as eulaminate (“well-laminated”), and are characterized by a visible layer IV. They show increasingly clear differentiation between layers II and III and in many areas between layers V and VI. Among eulaminate areas, koniocortex includes the most sharply laminated cortices, only seen in sensory areas. The primary motor cortex, as a specialized efferent system, has a distinctive layer V, and a small layer IV, but is strongly myelinated, like the well-laminated sensory association cortices [reviewed in García-Cabezas and Barbas (2014)]. We found that dimension 1 of the MDS reflects the discrete levels of lamination (Figures 2A,B). The limbic cortices were clustered near one end of Dimension 1 and the eulaminate cortices were clustered near the opposite end. This was reflected in the anticorrelation between Dimension 1 and the level of lamination (correlation coefficient = −0.61, *p* < 0.05). To further assess the ability of the MDS dimensions to predict the level of lamination, we performed partial correlation analysis, adding two additional features: the average levels of myelin and SMI-32. The partial correlation coefficients of the level of lamination with respect to five quantities—the three MDS dimensions and the average myelin, average SMI-32—were, respectively, −0.28 (*p* < 0.05), 0.24 (*p* < 0.05), −0.14, −0.08, and −0.17. Thus, the first two MDS dimensions reflected the level of lamination even after factoring out the average levels of myelin and SMI-32, indicating that the method extracted richer information than simply the average darkness of the stain.

We plotted the same MDS results according to cortical lobe or sub-system, which highlighted the fact that each sub-system contained its own structural spectrum (Figure 4). The prefrontal, motor, parietal, temporal, occipital, and insular sub-systems each showed a spectrum or gradient that aligned with the degree of lamination, which corresponded primarily with Dimension 1. The motor cortex spectrum aligned most closely with the assigned levels, followed by the temporal cortex spectrum. The other sub-systems showed more mixing or ambiguity among cortical areas, while preserving a general limbic-to-eulaminate gradation. Interestingly, while the occipital limbic-to-eulaminate gradation is reflected in Dimension 1, it is more clearly visible along Dimension 2. As mentioned above, partial correlation analysis indicates that the first two MDS dimensions do not simply reflect mean myelin and SMI-32 levels. Given that other quantifiable features cannot easily be extracted from the dataset, further comment on the nature of the implicit features being extracted is not straightforward, but may inform future research.

**FIGURE 6:**
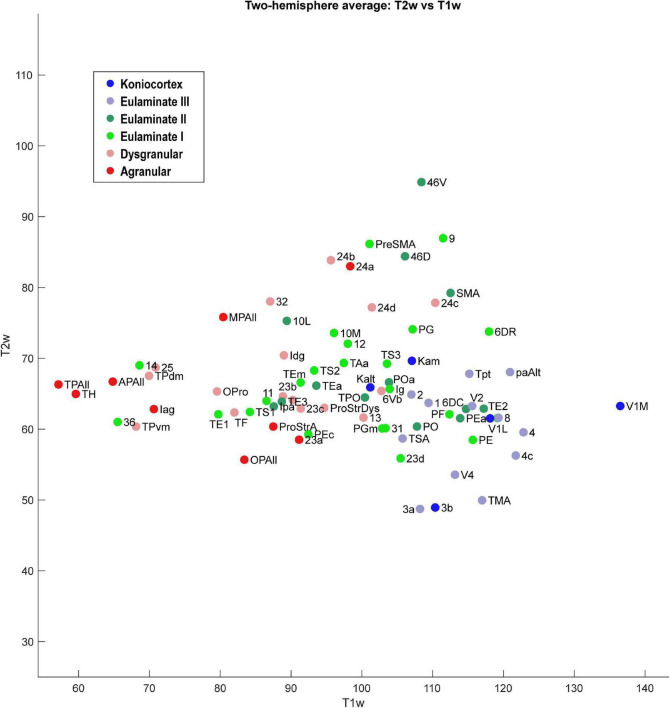
Scatter plot of 78 cortical areas. X-axis is T1-weighted MRI data, and Y-axis is T2-weighted MRI data. Each data point is the average of the optical density value for each area from the left and right hemisphere of the case analyzed.

**FIGURE 7:**
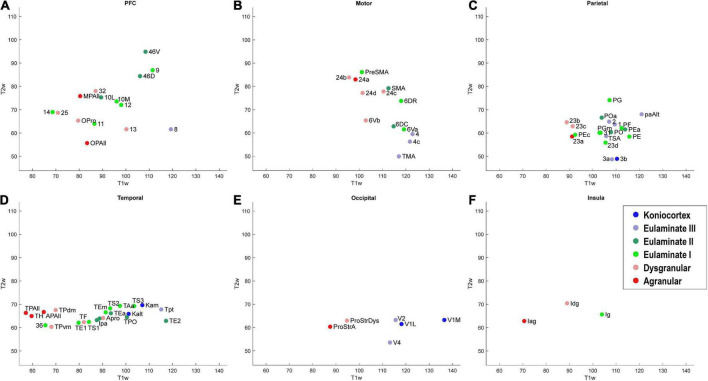
Scatter plot of T1w and T2w MRI data from 78 cortical areas, divided by system. **(A)** Prefrontal cortex, **(B)** Motor cortex, **(C)** Parietal cortex, **(D)** Temporal cortex, **(E)** Occipital cortex, **(F)** Insular cortex. Due to overlap of data points, area labels were omitted from some of the points for clarity.

We plotted the T1- and T2-weighted voxel values for each of the 78 cortical areas analyzed above, resulting in analogous scatter plots (Figures 6, 7). Due to the MRI resolution, we did not collect laminar data, but instead each cortical area produced a single mean gray value point of data per hemisphere. We averaged the data from the two hemispheres to produce a single two-dimensional dataset.

As seen in Figure 6, the MRI data capture the limbic-to-eulaminate trend seen in Dimension 1 of the MDS analysis. Specifically, the T1-weighted data is most informative of the degree of lamination. To facilitate further comparison with the MDS method, we also plotted the MRI data according to sub-system in Figure 7, which can be contrasted with Figure 4.

**FIGURE 8:**
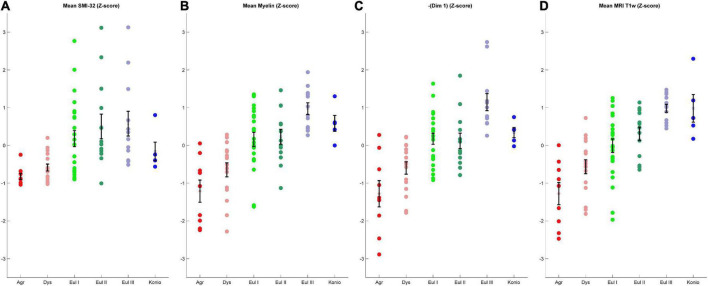
Comparison of four coarse-grained measurements that reflect the limbic-to-eulaminate spectrum. **(A)** Mean SMI-32 RGL. **(B)** Mean myelin RGL. **(C)** Sign-flipped MDS Dimension 1. **(D)** T1-weighted data. All data are z-scored. Error bars show standard error of the mean.

To assess the quality of analyses based on cortical profiles and MRI, we grouped the cortices by the expert-labeled cortical type and plotted the corresponding z-scored values of mean relative gray level (RGL) in SMI-32 images (Figure 8A); mean RGL in Gallyas images (Figure 8B); the negative of Dimension 1 from the MDS analysis of cortical profiles (Figure 8C); and T1-weighted MRI values (Figure 8D). Note that the MDS dimensions are arbitrarily set by the algorithm, and in this case the limbic-to-eulaminate direction was anti-correlated with MDS Dimension 1, so for comparison the sign was reversed. It can be seen that the cortical profile-based data (Figure 8C) and the T1-weighted MRI data both reflect the limbic-to-eulaminate spectrum. MDS analysis of each case, without averaging, produced broadly similar patterns (Supplementary Figures 4, 5). We also performed PCA on the same dataset and obtained qualitatively similar results (Supplementary material).

## Discussion

We showed that cortical profiles produced by analysis of cortical areas stained for myelin and SMI-32 support a conception of the cortical quilt as a structural gradient, which we refer to as the cortical spectrum. The spectrum derived from these cortical profiles shows broad agreement with cortical typologies derived from Nissl-stained cortical tissue, despite being performed without hand-labeling of layer boundaries. The Nissl stain has made it possible to identify cortical layers using transitions in features such as neuron arrangement and density. Once layer boundaries have been determined in cortical areas, the areas can be ordered according to the number of layers and the sharpness of transitions between layers, a pair of linked features which we refer to collectively as the degree of lamination. The degree of lamination is a key organizing principle of the cortex, as it enables prediction of the laminar pattern of origin and termination of cortico-cortical connections, their strength, and even presence or absence (Barbas, 1986; Barbas and Rempel-Clower, 1997; Medalla and Barbas, 2006; Hilgetag and Grant, 2010; Hilgetag et al., 2016; Goulas et al., 2018; García-Cabezas et al., 2019). Quantitative estimates of the degree of lamination are therefore needed.

**FIGURE 9:**
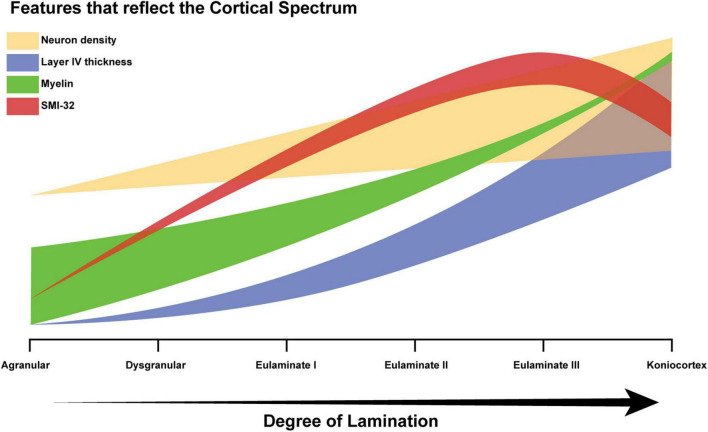
Schematic diagram of the cortical spectrum. The X-axis represents increasing degree of lamination, which corresponds to the traversal from agranular areas to koniocortical areas. The Y-axis represents the relative magnitudes of the features that serve as partial proxies of degree of lamination (arbitrary units). The thick translucent bar surrounding each plot corresponds to the variability in the feature. For example, the green bar corresponding to the variability in myelin density is initially thick and then becomes thinner, because a few agranular areas show high myelin, whereas, eulaminate areas are always well-myelinated. Limbic areas generally have low myelin content, but cortical myelin shows not only the intrinsic myelin of an area but also terminations from pathways from other structures that may be myelinated.

Neuron density, estimated from the widely used Nissl stain, serves as a partial proxy for degree of lamination, but is not suitable for all areas (García-Cabezas and Barbas, 2014). Another example of a Nissl-derived estimate is “externopyramidalization,” which is the ratio of soma sizes of supragranular pyramidal neurons (above layer IV) to those of infragranular pyramidal neurons (below layer IV) that correlates well with gradual cytoarchitectonic changes of the cortical mantle (Sanides, 1970; Pandya et al., 1988; Goulas et al., 2018; García-Cabezas et al., 2020). None of the quantitative features thus far identified is an ideal proxy for the assessment of an expert, but as such features are accumulated, the key concept becomes easier to recognize: cortical structure varies in a systematic and graded manner, resulting in a spectrum from limbic to eulaminate cortices. Figure 9 schematically represents how some of the feature gradients seen in the literature, including the present study, reflect degree of lamination. The thickness of each plot schematically reflects variability among cortical areas. For example, myelin roughly correlates with degree of lamination, but is high in some limbic areas, due to extensive myelinated axon projections from the thalamus. Similarly, the thickness of the neural density plot indicates that for some eulaminate cortices, density is a poor proxy: the primary motor cortex is an example, given that it is eulaminate but has relatively low neural density, due to the large size of its neurons, and in particular in layer V (García-Cabezas and Barbas, 2014). The SMI-32 plot is indicative of the fact that most well-laminated cortices have higher levels of SMI-32 than cortices that are relatively less well-laminated, as indicated in the data presented here (Figure 8A). The downward deflection toward koniocortex may arise because SMI-32 also tends to mark out neurons that send long distance projections (Campbell and Morrison, 1989), which koniocortices typically lack.

Data derived from Nissl-stained tissue provide useful proxies for degree of lamination, but their estimation tends to be a labor-intensive process. For example, estimating neural density requires examining cortical slices under a microscope to identify and categorize cells to enable stereological estimation of the number and density of neurons or distinct cell types (Pakkenberg and Gundersen, 1997). For this reason, the Nissl-derived quantitative estimates of degree of lamination have only been used in specific sub-systems of the cortex. The method described here, by contrast, produces a coarse but relatively rapid bird’s eye perspective of structural variation across the entire cortical mantle. Moreover, the technique can be applied to existing databases of cortical photomicrographs, and can be scaled up for comparison across species as well as among individuals within a species.

Our results confirm and extend prior observations that myelin and SMI-32 reveal architectonic features that serve as partial proxies for the degree of lamination (García-Cabezas and Barbas, 2014; García-Cabezas et al., 2017). In the case of myelin, the key feature is the extent to which myelin “reaches up” from below layer VI and into the gray matter. Note that myelin labels both efferent and afferent pathways. Efferent pathways from limbic areas are the least myelinated among cortical areas, but afferent fibers to limbic areas vary in myelin content depending on origin. In the case of SMI-32, the key features are (1) the presence of labeling in the deep part of layer III, and (2) the presence of labeling in layers V and VI. The method presented here enables a computational estimation of these qualitative features, which can be discerned in normalized cortical profiles derived from photomicrographs of the stained tissue. Dimension 1 of the MDS-space closely mirrors the subjectively assigned cortical levels.

Importantly, our method does not involve manual parcelation of the cortex into layers, and therefore partly mitigates experimenter variability when deciding on criteria for layer boundaries. The broad agreement with Nissl-derived typologies can therefore be interpreted as providing support for the qualitative approach. Some bias can in principle arise during the image thresholding procedure that reduces noise and enhances signal, but we attempted to minimize this possibility by using a blind and randomized approach, and averaging across experimenters, who were not experts. Nevertheless, the method is limited by the inherent coarseness of the granularity: features derived from image analysis are less precise than features extracted using more time-consuming microscropy and stereology. We view the method as a relatively rapid and exploratory view of cortical structure.

In humans, MRI is the primary means of studying structure non-invasively. Our results show that the cortical spectrum can also be discerned in T1-weighted MRI scans, which suggests a way to interpret this class of data. The large size of the human brain implies a higher-dimensional dataset than is possible in rhesus macaques for a given magnetic field strength, since multiple voxels will be available for each cortical area. Thus, the method outlined here—comparing cortical profiles using dimensionality reduction techniques such as MDS—may be well-suited to human MRI analyses. Given the widespread use of MRI, the cortical spectrum can be a powerful lens with which to contrast human brains in healthy and disordered subpopulations, and also to study individual differences.

In recent years, improvements in the resolution of MRI have enabled researchers to begin probing laminar structure, which is also described using terms such as microscale organization (van den Heuvel et al., 2015), cortical microstructure (Huntenburg et al., 2018), and microcircuits or microanatomy [Burt et al., 2018; reviewed in García-Cabezas et al. (2019)]. Neuroimaging data are already adding to the body of evidence in support of a central conclusion of the Structural Model: that structure predicts connectivity in the human brain (Zikopoulos et al., 2018; Paquola et al., 2020). The concept of a cortical spectrum can serve as a multi-level unifying principle, as it links low-dimensional structural gradients (Goulas et al., 2018, 2021; García-Cabezas et al., 2019, 2020) with the higher-dimensional intricacies of laminar elaboration, which in turn predicts connectivity (Barbas, 1986; Barbas and Rempel-Clower, 1997), and therefore sheds light on functional interaction among cortical areas (Huntenburg et al., 2017; Vázquez-Rodríguez et al., 2019; Paquola et al., 2020). For example, a gradient of functional connectivity appears to correlate with the cortical spectrum (Margulies et al., 2016). Specifically, the default mode network shows significant overlap with what we describe as the limbic end of the spectrum, furthest from primary sensory and motor cortices. Moreover, the cortical spectrum generates testable hypotheses about neurodevelopment (Dombrowski et al., 2001; Barbas and García-Cabezas, 2016; García-Cabezas et al., 2019; Barbas and Hilgetag, 2022), which in recent years has become grounded in causal gradients of gene expression organizing factors (Puelles and Ferran, 2012; Puelles et al., 2019). Specifically, the continuous gradation of degree of lamination informs the search for the underlying genetic factors that bring about these gradations. In contrast to measurements such as thickness (Wagstyl et al., 2020) or “distance” (Ercsey-Ravasz et al., 2013), this type of chemoarchitectonic patterning, which is reflected in the cortical spectrum, represents a more reliable lens with which to view cortical variation.

## Data availability statement

The raw data supporting the conclusions of this article will be made available by the authors, without undue reservation. The data and code used in this study are available at the following URL: https://www.bu.edu/neural/downloads1/.

## Ethics statement

The animal study was reviewed and approved by National Institutes of Health Guide for the care and use of laboratory animals (publication 80–22 revised, 1996) Institutional Animal Care and Use Committee at Boston University School of Medicine, Harvard Medical School, and New England Primate Research Center.

## Author contributions

YJ: formal analysis and software. HB and BZ: funding acquisition and resources. YJ, BZ, and MG-C: methodology. YJ and HB: writing. All authors conceptualization and approved the submitted version.

## Footnotes

1
http://fcon_1000.projects.nitrc.org/indi/PRIME/mcgill.html

## References

[B1] Barbas H. (1986). Pattern in the laminar origin of corticocortical connections. *J. Comp. Neurol.* 252 415–422. 10.1002/cne.902520310 3793985

[B2] Barbas H. (2015). General cortical and special prefrontal connections: Principles from structure to function. *Annu. Rev. Neurosci.* 38 269–289. 10.1146/annurev-neuro-071714-033936 25897871

[B3] Barbas H. García-Cabezas M. Á (2016). How the prefrontal executive got its stripes. *Curr. Opin. Neurobiol.* 40 125–134. 10.1016/j.conb.2016.07.003 27479655 PMC5056826

[B4] Barbas H. Hilgetag C. C. (2022). From circuit principles to human psychiatric disorders. *Biol. Psychiatry* in press. 10.1016/j.biopsych.2022.08.007 36114040

[B5] Barbas H. Rempel-Clower N. (1997). Cortical structure predicts the pattern of corticocortical connections. *Cereb. Cortex* 7 635–646. 9373019 10.1093/cercor/7.7.635

[B6] Bock N. A. Kocharyan A. Liu J. V. Silva A. C. (2009). Visualizing the entire cortical myelination pattern in marmosets with magnetic resonance imaging. *J. Neurosci. Meth.* 185 15–22. 10.1016/j.jneumeth.2009.08.022 19737577 PMC2783340

[B7] Brodmann K. (1909). *Vergleichende Lokalisationslehre der Grosshirnrinde in ihren Prinzipien dargestellt auf Grund des Zellenbaues.* Leipzig: Barth.

[B8] Burt J. B. Demirtaş M. Eckner W. J. Navejar N. M. Ji J. L. Martin W. J. (2018). Hierarchy of transcriptomic specialization across human cortex captured by structural neuroimaging topography. *Nat. Neurosci.* 21 1251–1259. 10.1038/s41593-018-0195-0 30082915 PMC6119093

[B9] Cahalane D. J. Charvet C. J. Finlay B. L. (2012). Systematic, balancing gradients in neuron density and number across the primate isocortex. *Front. Neuroanat.* 6:28. 10.3389/fnana.2012.00028 22826696 PMC3399120

[B10] Campbell A. W. (1905). *Histological studies on the localisation of cerebral function.* Sacramento, CA: Creative Media Partners, LLC.

[B11] Campbell M. J. Morrison J. H. (1989). Monoclonal antibody to neurofilament protein (SMI-32) labels a subpopulation of pyramidal neurons in the human and monkey neocortex. *J. Comp. Neurol.* 282 191–205. 10.1002/cne.902820204 2496154

[B12] Carlo C. N. Stevens C. F. (2013). Structural uniformity of neocortex, revisited. *Proc. Natl. Acad. Sci. U.S.A.* 110 1488–1493. 23297199 10.1073/pnas.1221398110 PMC3557031

[B13] Collins C. E. Airey D. C. Young N. A. Leitch D. B. Kaas J. H. (2010). Neuron densities vary across and within cortical areas in primates. *Proc. Natl. Acad. Sci. U.S.A.* 107 15927–15932. 10.1073/pnas.1010356107 20798050 PMC2936588

[B14] Dombrowski S. M. Hilgetag C. C. Barbas H. (2001). Quantitative architecture distinguishes prefrontal cortical systems in the rhesus monkey. *Cereb.Cortex* 11 975–988. 10.1093/cercor/11.10.975 11549620

[B15] Ercsey-Ravasz M. Markov N. T. Lamy C. Van Essen D. C. Knoblauch K. Toroczkai Z. (2013). A Predictive Network Model of Cerebral Cortical Connectivity Based on a Distance Rule. *Neuron* 80 184–197. 10.1016/j.neuron.2013.07.036 24094111 PMC3954498

[B16] García-Cabezas M. Á Barbas H. (2014). Area 4 has layer IV in adult primates. *Eur. J. Neurosci.* 39 1824–1834. 10.1111/ejn.12585 24735460 PMC4201116

[B17] García-Cabezas M. Á Hacker J. L. Zikopoulos B. (2020). A Protocol for Cortical Type Analysis of the Human Neocortex Applied on Histological Samples, the Atlas of Von Economo and Koskinas, and Magnetic Resonance Imaging. *Front. Neuroanat.* 14:576015. 10.3389/fnana.2020.576015 33364924 PMC7750391

[B18] García-Cabezas M. Á Joyce M. K. P. John Y. J. Zikopoulos B. Barbas H. (2017). Mirror trends of plasticity and stability indicators in primate prefrontal cortex. *Eur. J. Neurosci.* 46 2392–2405. 10.1111/ejn.13706 28921934 PMC5656436

[B19] García-Cabezas M. Á Zikopoulos B. Barbas H. (2019). The Structural Model: A theory linking connections, plasticity, pathology, development and evolution of the cerebral cortex. *Brain Struct. Funct.* 224 985–1008. 10.1007/s00429-019-01841-9 30739157 PMC6500485

[B20] Glasser M. F. Coalson T. S. Robinson E. C. Hacker C. D. Harwell J. Yacoub E. (2016). A multi-modal parcellation of human cerebral cortex. *Nature* 536 171–178. 10.1038/nature18933 27437579 PMC4990127

[B21] Goulas A. Changeux J.-P. Wagstyl K. Amunts K. Palomero-Gallagher N. Hilgetag C. C. (2021). The natural axis of transmitter receptor distribution in the human cerebral cortex. *Proc. Natl. Acad. Sci.* 118:e2020574118. 10.1073/pnas.2020574118 33452137 PMC7826352

[B22] Goulas A. Zilles K. Hilgetag C. C. (2018). Cortical Gradients and Laminar Projections in Mammals. *Trends Neurosci.* 41 775–788. 10.1016/j.tins.2018.06.003 29980393

[B23] Herculano-Houzel S. Watson C. R. Paxinos G. (2013). Distribution of neurons in functional areas of the mouse cerebral cortex reveals quantitatively different cortical zones. *Front. Neuroanat.* 7:35. 10.3389/fnana.2013.00035 24155697 PMC3800983

[B24] Hilgetag C. C. Grant S. (2010). Cytoarchitectural differences are a key determinant of laminar projection origins in the visual cortex. *NeuroImage* 51 1006–1017. 10.1016/j.neuroimage.2010.03.006 20211270

[B25] Hilgetag C. C. Medalla M. Beul S. F. Barbas H. (2016). The primate connectome in context: Principles of connections of the cortical visual system. *NeuroImage* 134 685–702. 10.1016/j.neuroimage.2016.04.017 27083526 PMC5135480

[B26] Hof P. R. Nimchinsky E. A. Morrison J. H. (1995). Neurochemical phenotype of corticocortical connections in the macaque monkey: Quantitative analysis of a subset of neurofilament protein-immunoreactive projection neurons in frontal, parietal, temporal, and cingulate cortices. *J. Comp. Neurol.* 362 109–133. 10.1002/cne.903620107 8576425

[B27] Horton J. C. Adams D. L. (2005). The cortical column: A structure without a function. *Philos. Trans. R. Soc. Lond. B Biol. Sci.* 360 837–862. 10.1098/rstb.2005.1623 15937015 PMC1569491

[B28] Huntenburg J. M. Bazin P.-L. Margulies D. S. (2018). Large-Scale Gradients in Human Cortical Organization. *Trends Cogn. Sci.* 22 21–31. 10.1016/j.tics.2017.11.002 29203085

[B29] Huntenburg J. M. Bazin P.-L. Goulas A. Tardif C. L. Villringer A. Margulies D. S. (2017). A Systematic Relationship Between Functional Connectivity and Intracortical Myelin in the Human Cerebral Cortex. *Cereb. Cortex* 27 981–997. 10.1093/cercor/bhx030 28184415 PMC5390400

[B30] Jana E. (2022). *Krippendorff’s Alpha*. MATLAB Central File Exchange. Available online at: https://www.mathworks.com/matlabcentral/fileexchange/36016-krippendorff-s-alpha (Accessed on Aug 24, 2022).

[B31] Large I. Bridge H. Ahmed B. Clare S. Kolasinski J. Lam W. W. (2016). Individual Differences in the Alignment of Structural and Functional Markers of the V5/MT Complex in Primates. *Cereb. Cortex* 26 3928–3944. 10.1093/cercor/bhw180 27371764 PMC5028002

[B32] Margulies D. S. Ghosh S. S. Goulas A. Falkiewicz M. Huntenburg J. M. Langs G. (2016). Situating the default-mode network along a principal gradient of macroscale cortical organization. *Proc. Natl. Acad. Sci. U.S.A.* 113 12574–12579. 10.1073/pnas.1608282113 27791099 PMC5098630

[B33] Medalla M. Barbas H. (2006). Diversity of laminar connections linking periarcuate and lateral intraparietal areas depends on cortical structure. *Eur. J. Neurosci.* 23 161–179. 10.1111/j.1460-9568.2005.04522.x 16420426

[B34] Pakkenberg B. Gundersen H. J. (1997). Neocortical neuron number in humans: Effect of sex and age. *J. Comp. Neurol.* 384 312–320. 9215725

[B35] Palomero-Gallagher N. Zilles K. (2018). Cyto- and receptor architectonic mapping of the human brain. *Handb. Clin. Neurol.* 150 355–387. 10.1016/B978-0-444-63639-3.00024-4 29496153

[B36] Pandya D. Seltzer B. Barbas H. (1988). Input-output organization of the primate cerebral cortex. *Neurosci. Comp. Primate Biol.* 4 39–80.

[B37] Paquola C. Seidlitz J. Benkarim O. Royer J. Klimes P. Bethlehem R. A. I. (2020). A multi-scale cortical wiring space links cellular architecture and functional dynamics in the human brain. *PLoS Biol.* 18:e3000979. 10.1371/journal.pbio.3000979 33253185 PMC7728398

[B38] Puelles L. Alonso A. García-Calero E. Martínez-de-la-Torre M. (2019). Concentric ring topology of mammalian cortical sectors and relevance for patterning studies. *J. Comp. Neurol.* 527 1731–1752. 10.1002/cne.24650 30737959

[B39] Puelles L. Ferran J. L. (2012). Concept of neural genoarchitecture and its genomic fundament. *Front. Neuroanat.* 6:47. 10.3389/fnana.2012.00047 23181010 PMC3499902

[B40] Rockel A. J. Hiorns R. W. Powell T. P. (1980). The basic uniformity in structure of the neocortex. *Brain* 103 221–244. 10.1093/brain/103.2.221 6772266

[B41] Sanides F. (1970). “Functional architecture of motor and sensory cortices in primates in the light of a new concept of neocortex evolution,” in *The Primate Brain: Advances in Primatology*, eds Noback C. R. Montagna W. (New York, NY: Appleton-Century-Crofts Educational Division)137–208.

[B42] van den Heuvel M. P. Scholtens L. H. Feldman Barrett L. Hilgetag C. C. de Reus M. A. (2015). Bridging Cytoarchitectonics and Connectomics in Human Cerebral Cortex. *J. Neurosci.* 35 13943–13948. 10.1523/JNEUROSCI.2630-15.2015 26468195 PMC6608182

[B43] Vázquez-Rodríguez B. Suárez L. E. Markello R. D. Shafiei G. Paquola C. Hagmann P. (2019). Gradients of structure–function tethering across neocortex. *Proc. Natl. Acad. Sci. U.S.A.* 116 21219–21227. 10.1073/pnas.1903403116 31570622 PMC6800358

[B44] Vickery S. Hopkins W. D. Sherwood C. C. Schapiro S. J. Latzman R. D. Caspers S. (2020). Chimpanzee brain morphometry utilizing standardized MRI preprocessing and macroanatomical annotations. *eLife* 9:e60136. 10.7554/eLife.60136 33226338 PMC7723405

[B45] von Economo C. (1927). *Zellaufbau der Grosshirnrinde des Menschen: Zehn Vorlesungen.* Riga: VDM Verlag Dr. Müller.

[B46] von Economo C. Koskinas G. N. Triarhou L. C. (1925). *Atlas of Cytoarchitectonics of the Adult Human Cerebral Cortex, Vol. 10.* Basel: Karger Basel.

[B47] Wagstyl K. Larocque S. Cucurull G. Lepage C. Cohen J. P. Bludau S. (2020). BigBrain 3D atlas of cortical layers: Cortical and laminar thickness gradients diverge in sensory and motor cortices. *PLoS Biol.* 18:e3000678. 10.1371/journal.pbio.3000678 32243449 PMC7159250

[B48] Zhang J. Scholtens L. H. Wei Y. van den Heuvel M. P. Chanes L. Barrett L. F. (2020). Topography Impacts Topology: Anatomically Central Areas Exhibit a “High-Level Connector”. Profile in the Human Cortex. *Cereb. Cortex* 30 1357–1365. 10.1093/cercor/bhz171 31504277 PMC7132940

[B49] Zikopoulos B. García-Cabezas M. Á Barbas H. (2018). Parallel trends in cortical gray and white matter architecture and connections in primates allow fine study of pathways in humans and reveal network disruptions in autism. *PLoS Biol.* 16:e2004559. 10.1371/journal.pbio.2004559 29401206 PMC5814101

