# Supplementary material for: The cortical spectrum: A robust structural continuum in primate cerebral cortex revealed by histological staining and magnetic resonance imaging
Source: Front Neuroanat. 2022 Sep 9;16:897237. doi: 10.3389/fnana.2022.897237 (PMC9501703; doi:10.3389/fnana.2022.897237)
Supplement: Supplementary file 1 [file Data_Sheet_1.docx]

**Supplementary Material**

In addition to the MDS analysis, we performed principal component analysis (PCA), using Matlab’s in-built PCA function. We found qualitatively similar results (Figure S1). As with MDS, Dimension 1 was the most predictive of the degree of lamination. The first three PCA dimensions explained 29.5%, 14.4%, and 8.1% of the variance, respectively. The first three PCA dimensions correlate closely with the MDS dimensions, showing correlation coefficients of 0.97, 0.97, and 0.99, respectively. Partial correlation analysis indicates that Dimension 1 captures the subjectively assessed degree of lamination even after accounting for mean myelin and SMI-32 levels. The partial correlation coefficients of the first three PCA dimensions and the mean myelin and SMI-32 levels were, respectively, -0.2666 (p<0.05), 0.2155, -0.1041, 0.1237, -0.1243. These results are comparable to those obtained from the MDS analysis.

The accuracy of the MDS dimensions for capturing inter-area differences was estimated using linear regression: the Euclidean distances (L2 norm) between each pair of cortical areas in MDS-space were regressed on the corresponding Euclidean distances computed from the original Z-scored dataset. The slope of the regression line was 1.05 and the adjusted R-squared value was 0.84, indicative of a good fit (Figure S2). Note that the dataset does not contain the spatial locations of the cortical areas in three-dimensional space. “Euclidean distance” here refers to the L2-norm used to computer differences between pairs of cortical areas.

If, instead of averaging, each case is analyzed separately, the qualitative results of MDS are similar (Figures S4, S5).


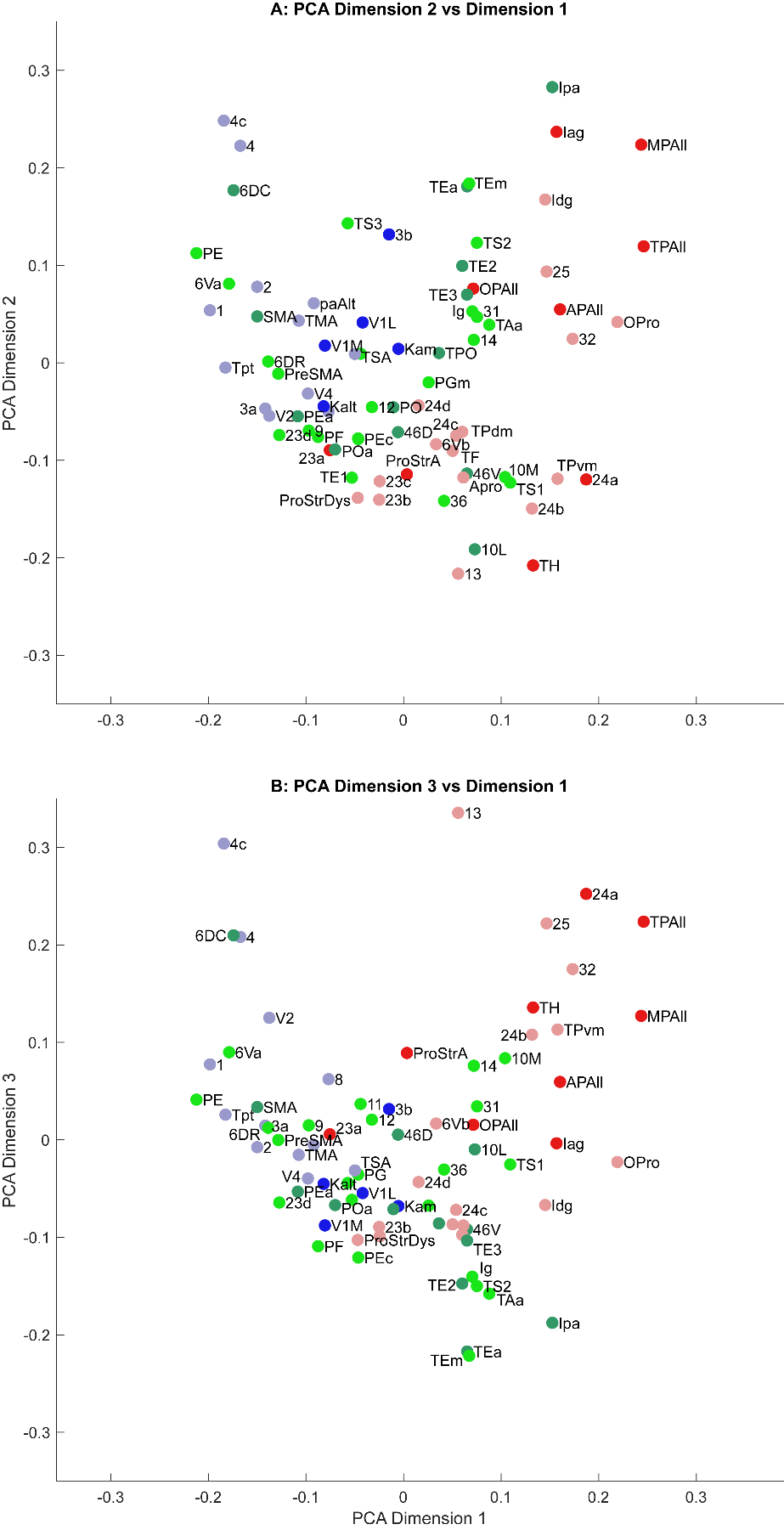


**Figure S1. Scatter plot of 78 cortical areas in PCA dimensions.** We show the first three PCA dimensions. Dimension 1 showed the clearest alignment with the subjective classification of cortical types according to degree of lamination. A. Dimension 2 versus Dimension 1. B. Dimension 3 versus Dimension 1. Due to partial overlap of data points, some area labels were omitted for clarity.


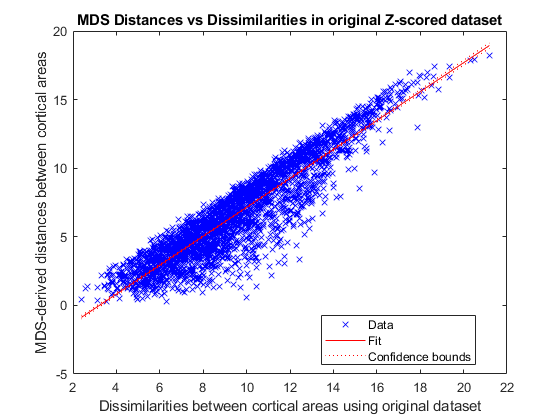


**Figure S2. Accuracy of MDS.** Linear regression showing that MDS accurately captures dissimilarities among cortical areas in the original Z-scored dataset. Adjusted R-squared = 0.84.


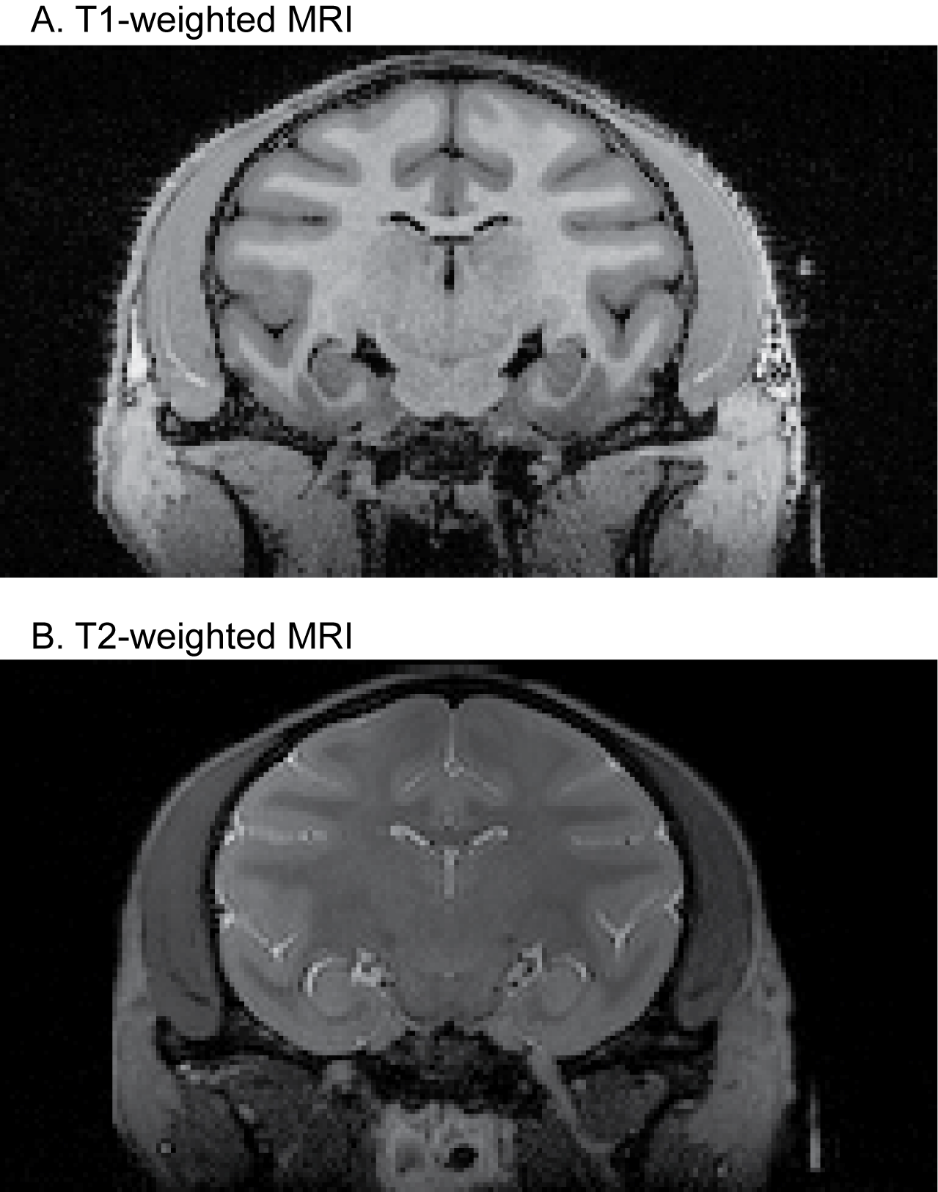


**Figure S3. Examples of MRI images**. A: T1-weighted MRI. B: T2-weighted MRI. The two images are matched for the z-direction. Voxel size: 0.6x0.6x0.6mm.


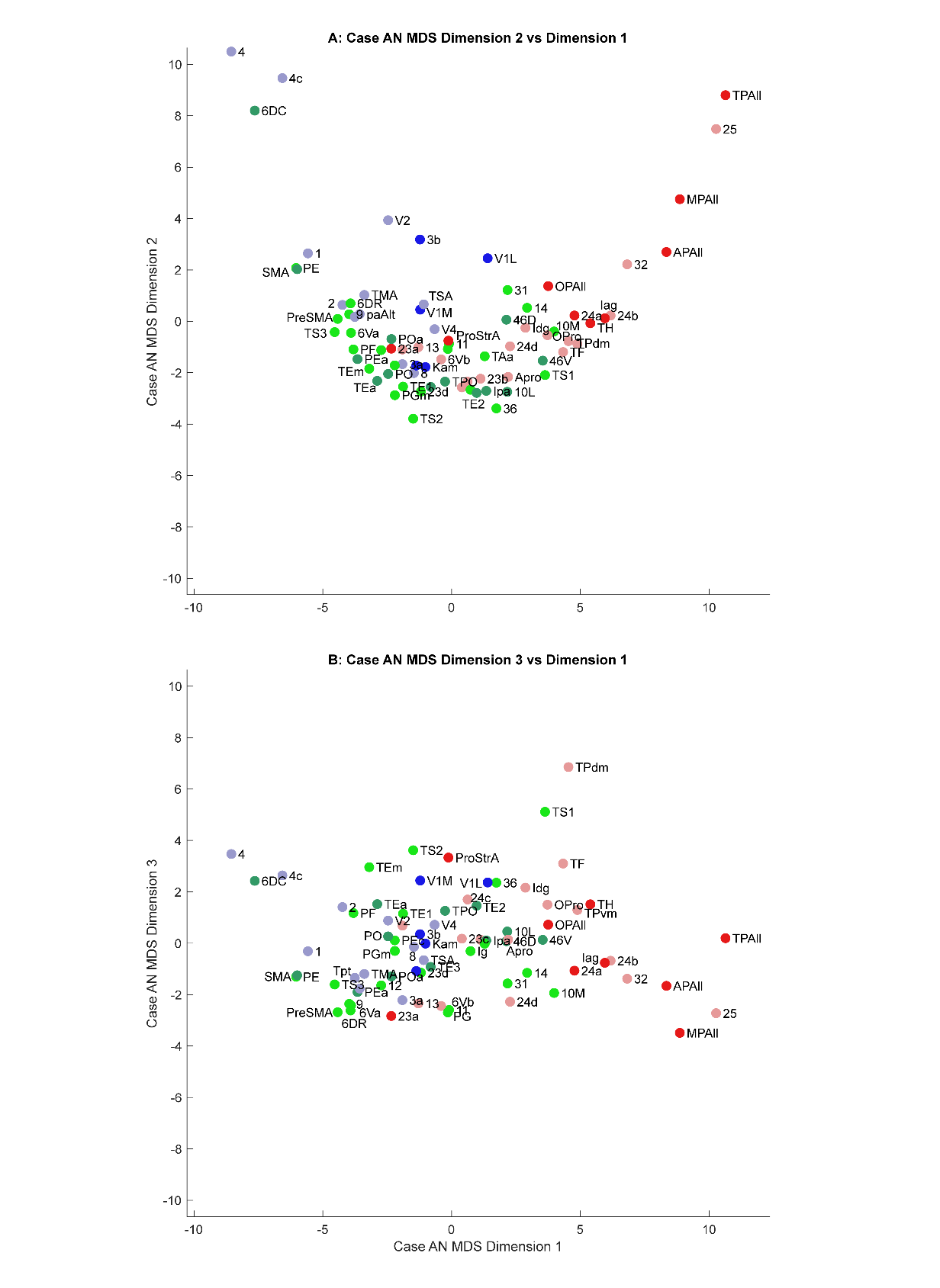


**Figure S4. Scatter plot of 78 cortical areas in MDS dimensions for case AN.** Analysis of a single case indicates that the qualitative results hold for individual cases. Due to partial overlap of data points, some area labels were omitted for clarity.


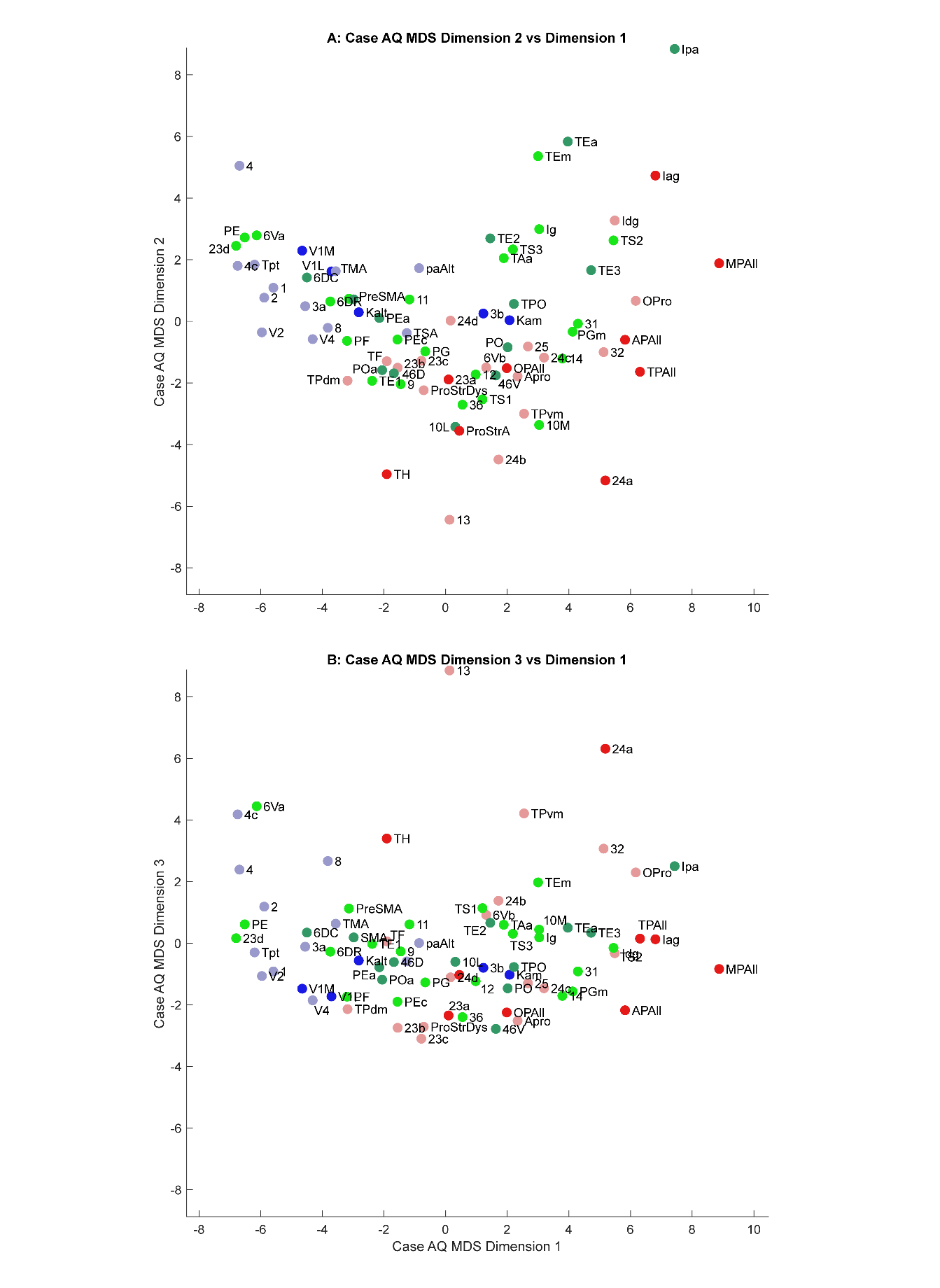


**Figure S5. Scatter plot of 78 cortical areas in MDS dimensions for case AQ.** Analysis of a single case indicates that the qualitative results hold for individual cases. Due to partial overlap of data points, some area labels were omitted for clarity.
